# P-51. Carbapenem-Resistant Pseudomonas aeruginosa Bloodstream Infections: A Retrospective Case-Control Study

**DOI:** 10.1093/ofid/ofaf695.280

**Published:** 2026-01-11

**Authors:** Osvaldo D Cabrera-Castellanos, Gabriel Cedeño-Sánchez, Francisco Guzman-Ricardo, Yeison Reyes-Burgos, Ann S Sánchez-Marmolejos, Anel E Guzmán-Marte, José A Ledesma-Baéz, Rubén Calcaño, Rita A Rojas-Fermín

**Affiliations:** Hospital General Plaza de la Salud, santo domingo, Distrito Nacional, Dominican Republic; Hospital general plaza de la salud, Santo Domingo, Distrito Nacional, Dominican Republic; Hospital General de la Plaza de la Salud, Santo Domingo, Distrito Nacional, Dominican Republic; Hospital General Plaza de la Salud, santo domingo, Distrito Nacional, Dominican Republic; Hospital General de la Plaza de la Salud, Santo Domingo, Distrito Nacional, Dominican Republic; Hospital General De La Plaza De La Salud, Distrito Nacional, Distrito Nacional, Dominican Republic; Hospital General De La Plaza De La Salud, Distrito Nacional, Distrito Nacional, Dominican Republic; Hospital General De La Plaza De La Salud, Distrito Nacional, Distrito Nacional, Dominican Republic; Hospital General De La Plaza De La Salud, Distrito Nacional, Distrito Nacional, Dominican Republic

## Abstract

**Background:**

*Pseudomonas aeruginosa* (PA) is an opportunistic pathogen linked to bacteremia and infections in healthcare settings. Carbapenem-resistant *P. aeruginosa* (CRPA) is classified as a high priority pathogen on the World Health Organization´s (WHO) priority list. Therefore, this study aims to characterize the microbiological and clinical patterns, risk factors and clinical manifestations of *P. aeruginosa* bacteremia in a hospital setting in the Dominican Republic.

**Graphic 1:**
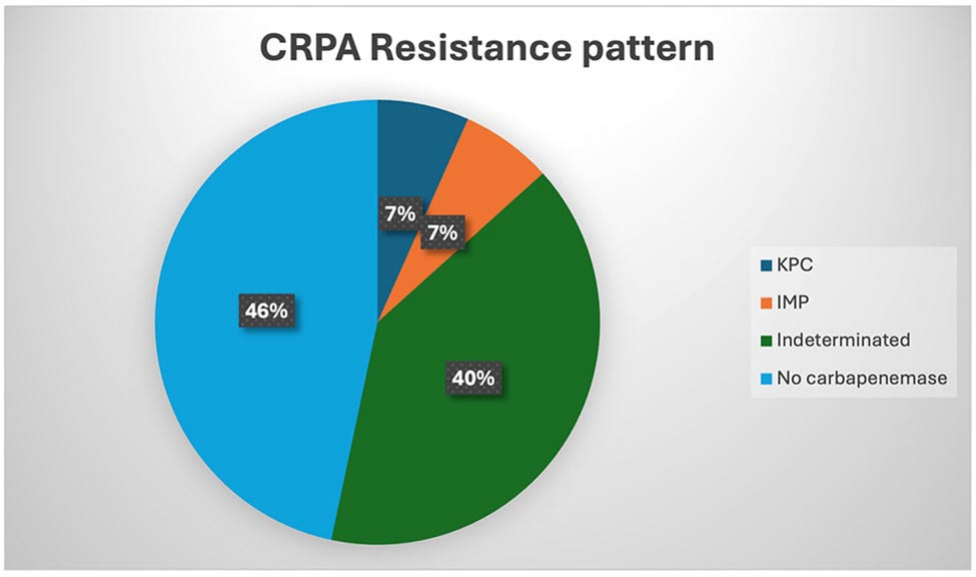
Resistance pattern in CRPA isolates (n = 15)

**Graphic 2:**
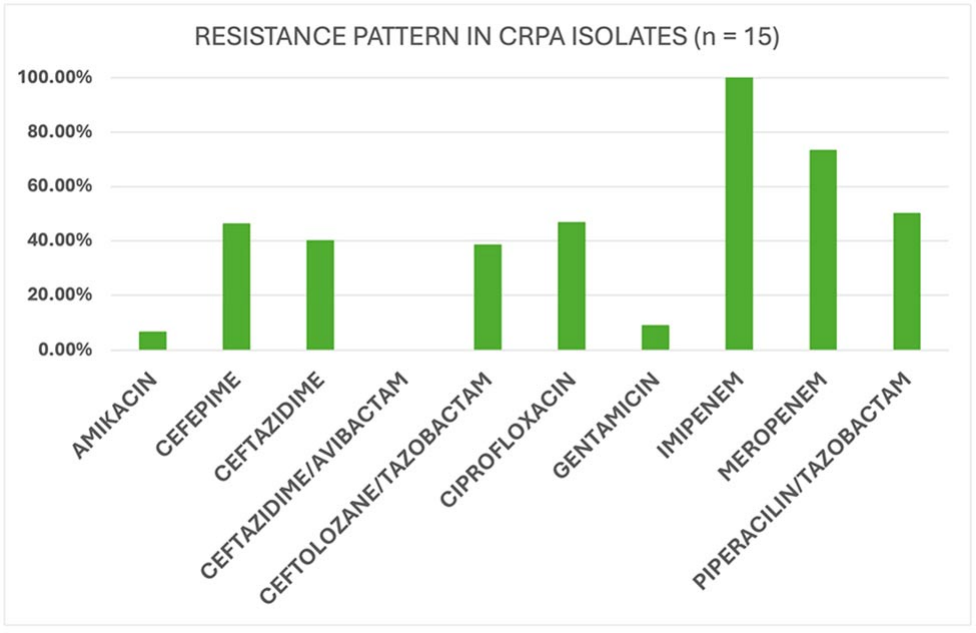
Antimicrobial resistance of CRPA isolates (n = 15)

**Methods:**

A retrospective case-control study was conducted in 79 patients with *P. aerugino*sa bacteremia (15 CRPA and 64 Carbapenem-susceptible) from March 2021 to November 2024. Electronic medical records were reviewed for demographics, co-morbidities, risk factors, resistance patterns, and outcomes. An analysis was performed to calculate Odds Ratio (OR) and p-value ≤ 0.05.

**Table 1:**
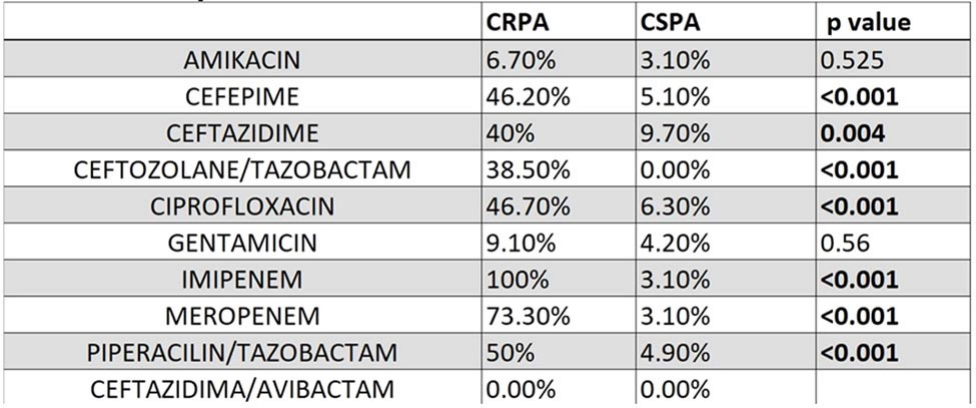
Comparison of Antibiotic Resistance Between CRPA and CSPA Isolates

**Graphic 3:**
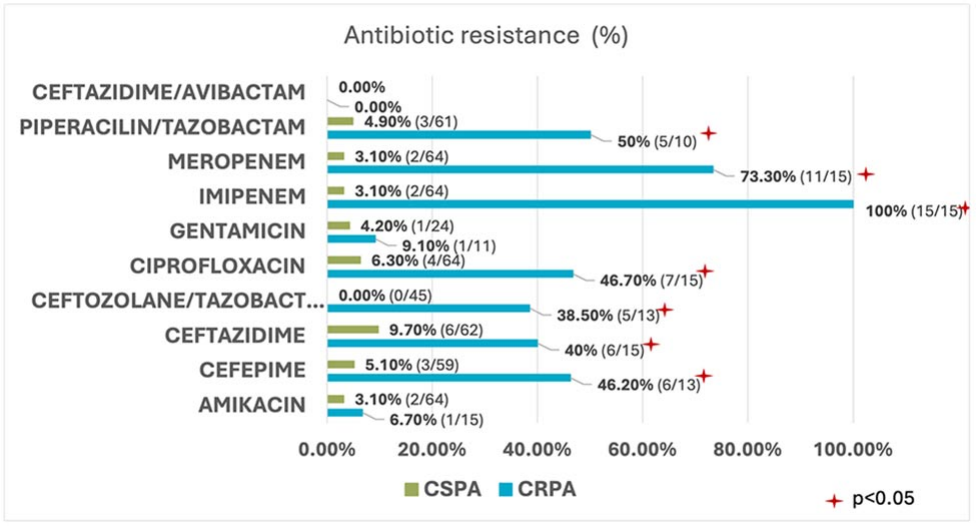
Antibiotic resistance in CRPA vs. CSPA isolates (%).

**Results:**

Carbapenemase production was negative (7/15), indeterminate (6/15) or positive (1/15 KPC and 1/15 IMP). Mortality in CRPA patients was at 46.7% (7/15) vs 37.5% (24/64) in CSPA. Regarding susceptibility patterns, resistance to Ceftolozane-Tazobactam was 38.5% (5/13; 2 missing values). In relation to ceftazidime-avibactam, 100% of isolates evaluated for resistance were susceptible (13/13; 2 missing values). Length of stay (LOS) of ≥15 days was identified as a potential risk factor for CRPA infection when compared to CSPA (p = 0.004). Notably, patients with CRPA infections exhibited a prolonged LOS.

**Conclusion:**

CRPA bloodstream infections are associated with high mortality, frequently attributed to carbapenemase production, carbapenemase identification remained indetermined in a significant proportion of isolates (40%) due to phenotypic testing limitations. Improved carbapenemase detection and enhanced antimicrobial stewardship and infection control are crucial to combat the high mortality and economic costs of CRPA bloodstream infections, especially in prolonged-stay ICU patients.

**Disclosures:**

Rita A. Rojas-Fermín, MD, GSK: Honoraria

